# Aggressive progression to EGFR tyrosine kinase inhibitors in advanced NSCLC patients: concomitant mutations, prognostic indicator and subsequent management

**DOI:** 10.1007/s00432-023-04757-4

**Published:** 2023-04-19

**Authors:** Ruishan Wen, Ying Chen, Jinyu Long, Xiulian Huang, Yuxin Guo, Baoquan Lin, Zongyang Yu

**Affiliations:** 1 College of Rehabilitation Medicine, Fujian University of Traditional Chinese Medicine, Fuzhou, China; 2 Department of Respiratory and Critical Care Medicine, The 900th Hospital of the Joint Logistic Support Force, People’s Liberation Army of China, 156 Xierhuan North Road, Fuzhou, 350025 China; 3 Fuzong Clinical Medical College, Fujian Medical University, Fuzhou, Fujian China; 4 Department of Cardio-Thoracic Surgery, The 900th Hospital of the Joint Logistic Support Force, People’s Liberation Army of China, 156 Xierhuan North Road, Fuzhou, 350025 China

**Keywords:** Non-small cell lung cancer, EGFR mutation, 19Del, L858R, Aggressive progression

## Abstract

**Background:**

EGFR tyrosine kinase (TKIs) are recommend as the first-line treatment for non-small cell lung cancer (NSCLC) patients with EGFR mutation. However, some patients experience aggressive progression with a progression-free survival (PFS) less than 6 months on the first-line EGFR TKI therapy. Therefore, our study is to analyze the potential influencing factors including clinical features, biomarkers, concomitant mutations et al.

**Methods:**

A total of 1073 NSCLC patients with EGFR mutation in a multi-center study from January 2019 to December 2021. The datum pathological and molecular characteristics were collected. The area under the receiver operating characteristic (ROC) curve was used to evaluate the predictive effect of Ki-67 on the first-line TKI. The curve of PFS was conducted by Kaplan–Meier method and tested by bilateral log-rank. Cox regression model was used to predict and evaluate PFS of different variables. Chi-square or Fisher analysis was used for correlation between groups.

**Results:**

55 patients who show aggressive progression (PFS ≤ 6 months) on the first-line TKI therapy were analyzed in this study, while 71 with slow progression (PFS > 6 months). Concomitant mutations including AXIN2, P2CG and RAD51C mutations occurred only in the aggressively progressive group (*P* = 0.029). Correlation between Ki-67 index and the aggressive progression of the first-line TKI therapy was significant statistically different (*P* < 0.05). In the second-line therapy, the PFS of chemotherapy in combination with other treatments was better than single TKIs in the first ten months.

**Conclusion:**

NSCLC harbored EGFR and concomitant mutations (such as AXIN2, PLCG2 and RAD51C), and/or Ki-67 high expression may indicate the aggressive progression to the first-line EGFR-TKI.

## Introduction

According to the International Agency for Research on Cancer, there will be 19.3 million new cases of malignant tumors and nearly 10 million deaths worldwide in 2020, with lung cancer accounting for 11.4% of the total new cases and 18% of the deaths (Sung et al. 2021). It is widely known that EGFR mutations are most common in East Asian, female, non-smoking patients with lung adenocarcinoma. As the two most major subtypes of EGFR mutations, the EGFR exon 19 deletion (19 del) and the exon 21 Leu858Arg (L858R) mutation account for 85–90% of all EGFR mutations (Lynch et al. 2004; Paez et al. 2004). The American Society of Clinical Oncology (ASCO) and National Comprehensive Cancer Network (NCCN) guidelines recommend EGFR tyrosine kinase (TKIs) as the first-line treatment for those EGFR-mutation-positive patients. Unfortunately, although EGFR TKIs provide a modest outcome, most patients will eventually develop progressive disease (PD). In clinical practice, we found that EGFR mutant patients have demonstrated heterogeneity in EGFR TKI treatment responses with progression-free survival (PFS). Some of them demonstrated an aggressive progression following EGFR TKI treatments, while others demonstrated response periods for several years.

In addition to the EGFR sensitizing mutation, Ki-67 index may affect the outcomes of TKI treatment. To explore the biomarker of primary resistance, we divided advanced NSCLC patients with EGFR 19 del or L858R mutations into two groups, one for aggressively progressive response and the other for slowly progressive response following first-line EGFR-TKI treatments. Pan-cancer gene next-generation sequencing was used for tumor genomic profiling.

## Methods

### Patients

From January 2019 to December 2021, among 1,073 pathologically confirmed lung carcinoma patients were enrolled and 55 eligible patients were included in this multi-center study (including The 900th Hospital of the Joint Logistic Support Force, PLA; Fujian Medical University Union Hospital; Fujian Provincial Hospital; The Second Affiliated Hospital of Fujian Medical University). Patients who met the following criteria were included in the analysis: (1) confirmed NSCLC by pathology; (2) PCR-based direct sequencing, next-generation sequencing 448 gene or 10 gene panel were used to detect the EGFR exon 19 deletion or L858R mutation(Amoy Dx NGS data analysis system ADXLC10 module was used for bioinformation analysis); (3) first-line treatment was EGFR TKI treatment; (4) the objective tumor response was measured according to the Response Evaluation Criteria in Solid Tumors version 1.1 (RECIST 1.1); and (5) complete clinical and first-line treatment data. Progression-free survival (PFS) was defined as time from the start of therapy to the date of disease progression or death.

The study was conducted in conformity to the Declaration of Helsinki (as revised in 2013). The study was approved by institutional ethics board of the 900th Hospital of the Joint Logistic Support Force, PLA (No. 2021-026) and all hospitals where the included patients are from have approved the study. Informed consent was taken from all the patients.

### Data collection

Clinical data including age, gender, smoking status, gene mutation, histologic type, IHC, pathological stage, the first-line therapy, and best response were collected by reviewing the medical charts and pathology records. The imaging data mainly including computed tomography (CT) scans and magnetic resonance imaging (MRI) scans, which were independently reviewed by the authors to evaluate their treatment responses according to RECIST 1.1. Cross-checking was performed for response evaluation to mitigate the problem of measurement errors. Types of response included complete response (CR), partial response (PR), stable disease (SD), or progressive disease (PD). The best response of each patient was recorded. Progression-free survival (PFS) was adopted as main therapeutic outcomes.

### Statistical analysis

Statistical analyses were performed with IBM SPSS Statistics 26.0 software. Chi-square or Fisher’s exact tests were used to compare qualitative data. *T*-test was conducted to analyze the correlation between the Ki-67 index and the response of the first-line TKI treatment. Kaplan–Meier method was used to estimate PFS curves, while log-rank test was used to estimate the survival curves among patient groups. The Cox regression model was introduced to identify the predictive effect on PFS of different variables. All statistical tests were two-sided and *P* < 0.05 was considered to be statistically significant. GraphPad Prism (GraphPad Inc., La Jolla, CA, USA) was used to generate survival curves and receiver operating characteristic (ROC) curves. and a complex heatmap was constructed.

## Results

### Clinicopathological characteristics

**Fig. 1:**
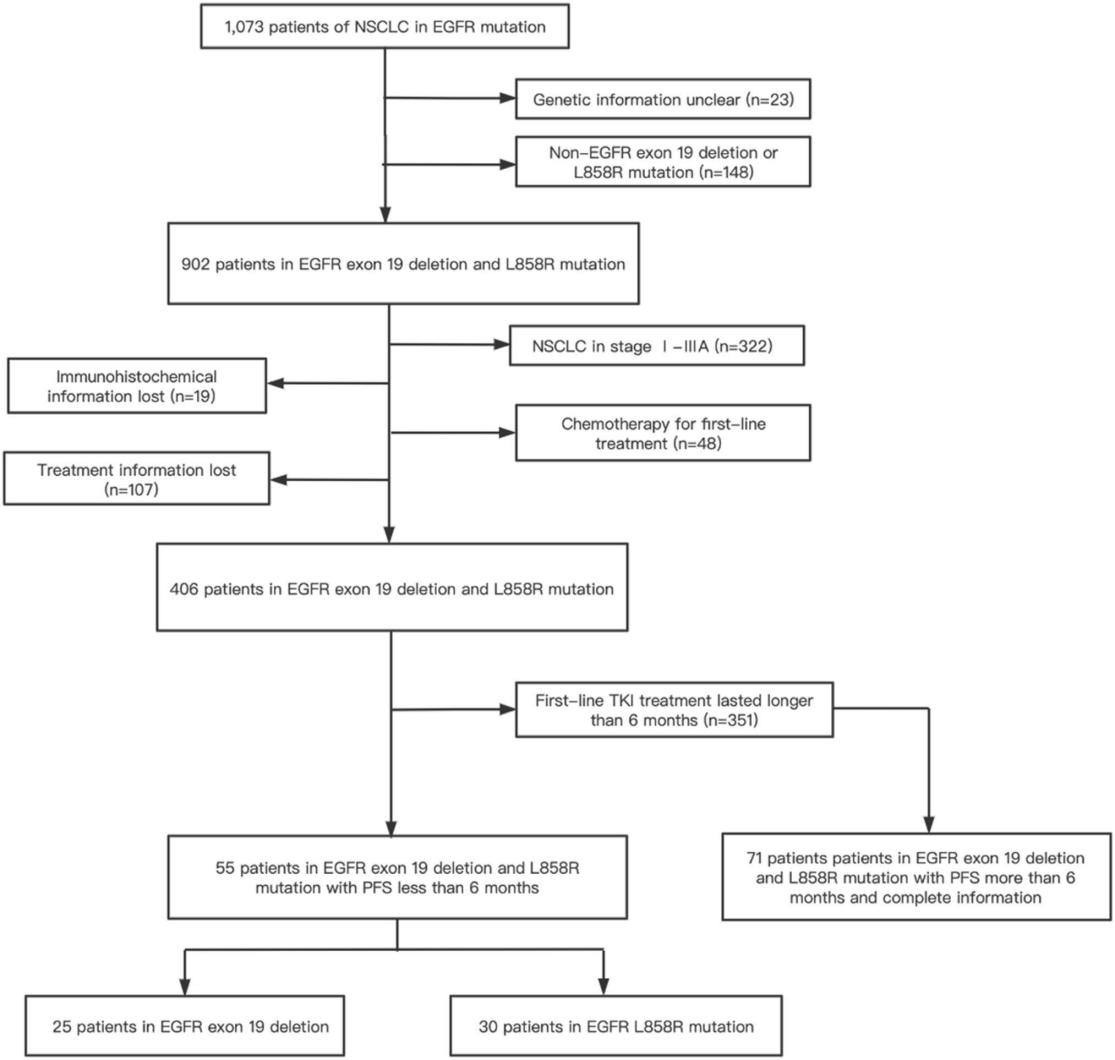
Flow chart of study design and patients enrollment

In our study, a total of 126 cases with advanced lung adenocarcinoma were retrospectively included (Fig. 1). All patients harbored either EGFR exon 19 deletion mutation or EGFR L858R mutation and had received the EGFR TKI treatment as the first-line treatment. As shown in Table 1, patients were divided into two groups: the aggressively progressive responders (*n* = 55) with a PFS of less than 6 months after first-line TKI treatment (mPFS: 4 months, range: 0.5–6 months) and the slowly progressive responders (*n* = 71) was extremely long PFS of over 6 months (mPFS: 11 months, range: 7–54 months).

**Table 1:** Clinicopathological parameters of patients

	Aggressively progressive group (*n* = 55)	Slowly progressive group (*n* = 71)	*P* value
*Age (years)*			0.069
< 65	33 (60%)	42 (59.2%)	
≥ 65	22 (40%)	29 (40.8%)	
*Gender*			0.680
Male	22 (40.0%)	31 (43.7%)	
Female	33 (60.0%)	40 (56.3%)	
*Smoking status*			0.584
Smokers	8 (14.5%)	8 (11.3%)	
Never	47 (85.5%)	63 (88.7%)	
*Histology*			0.396
Adenocarcinoma	53 (96.4%)	70 (98.6%)	
Squamous cell carcinoma	2 (3.6%)	1 (1.4%)	
*EGFR mutations*			0.065
L858R	30 (54.5%)	27 (38.0%)	
19 del	25 (45.5%)	44 (62.0%)	
*Basic diseases*			0.425
Yes	31 (56.4%)	45 (63.4%)	
No	24 (43.6%)	26 (36.6%)	
*Malignant pleural effusion*			0.715
Yes	23 (41.8%)	32 (45.1%)	
No	32 (58.2%)	38 (53.5%)	
*First-line EGFR TKIs*			0.057
1st-gen TKI	44 (80.0%)	54 (76.1%)	
2nd-gen TKI	1 (1.8%)	9 (12.7%)	
3rd-gen TKI	10 (18.2)	8 (11.3%)	

Among the patients in the aggressively progressive group, there were 22 males (40%) and 33 females (66.0%), and 22 (44.0%) were over 65 years old. Among them, 8 (14.5%) were smokers and 47 (85.5%) were non-smokers. According to histological type, 53 patients (96.4%) were diagnosed as adenocarcinoma and only 2 patients (3.6%) were diagnosed as squamous cell carcinoma. 30 (54.5%) patients harbored EGFR L858R mutation and 25 (45.5%) harbored EGFR 19 del mutation. Different generations of TKI were used as first-line treatment, including first-generation TKI in 80%, second-generation TKI in 1.8% and third-generation TKI in 18.2%. The best response was PR in 20%, SD in 36.4%, and PD in 27.3% at the first efficacy evaluation. We found that all features, such as age, sex, smoking status, histology, EGFR mutation, basic diseases, malignant pleural effusion and the first-line EGFR TKI were not significantly different between the two groups.

**Fig. 2:**
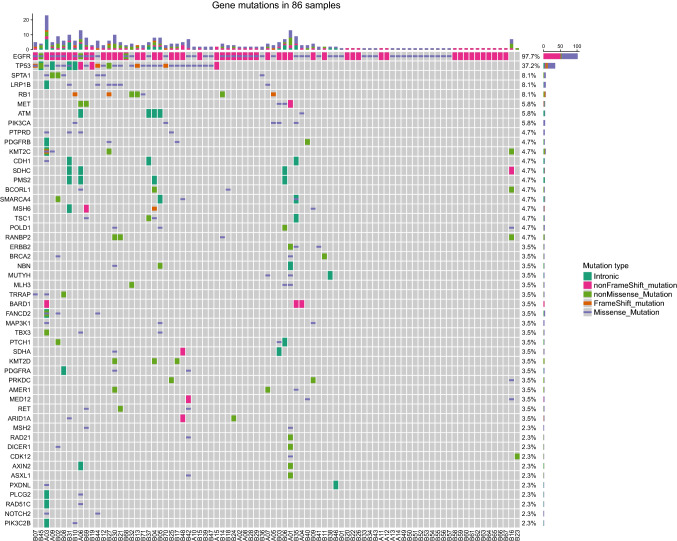
The OncoPrint depicts gene mutations in 86 samples. Mutation frequencies per gene and per subtype are shown here as the percentage of the total number of patients. EGFR, epidermal growth factor receptor; TP53, recombinant tumor protein 53; SPTA1, alpha spectrin; LRP1B, lipoprotein receptor-related protein 1B; RB1, retinoblastoma 1. **A** Patients in aggressively progressive group. **B** Patients in slowly progressive group

**Fig. 3:**
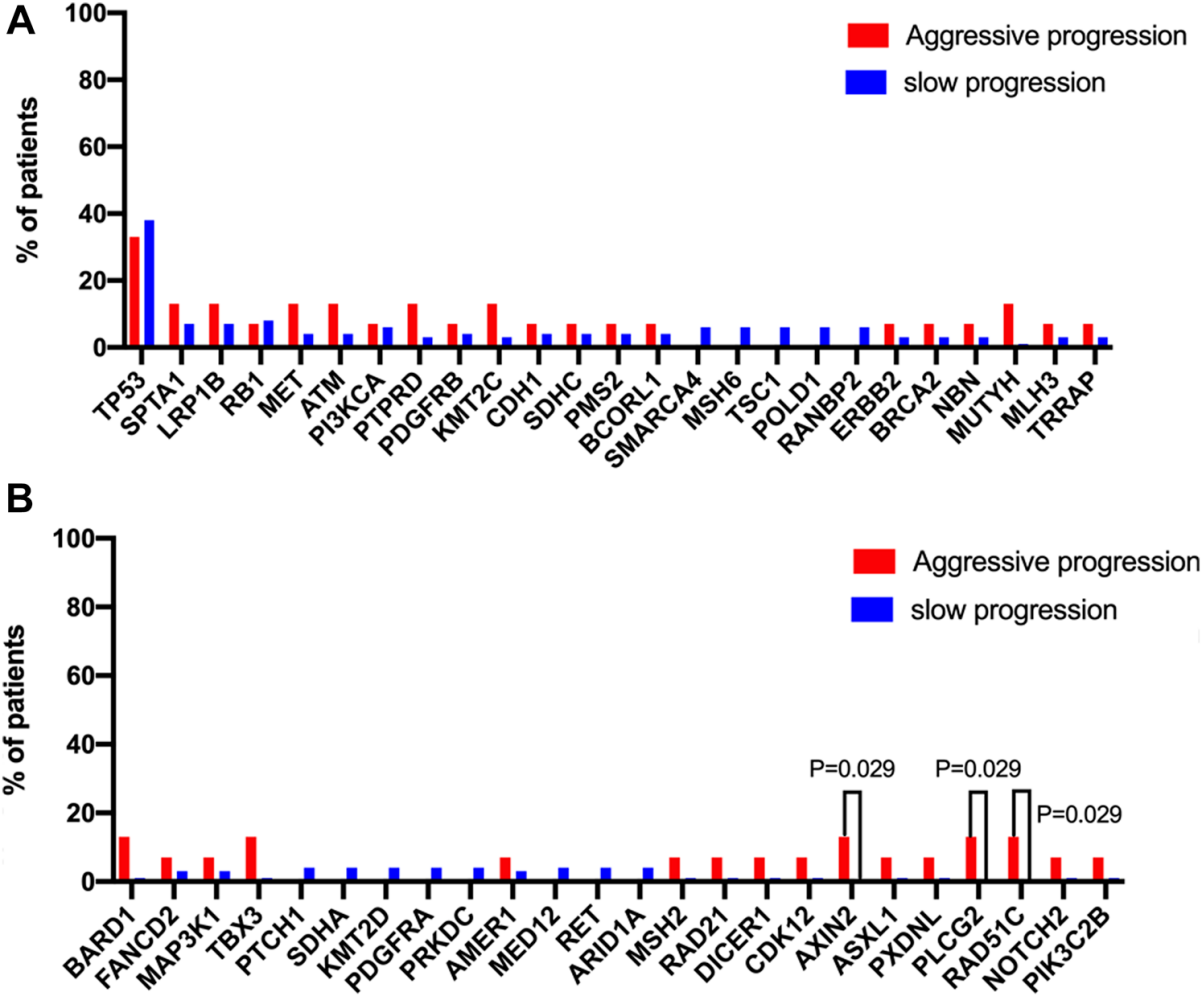
Comparison of the mutated genes in aggressively progressive and slowly progressive groups. The chi-squared test was used to compare the proportions of each group that contained the mutations listed

Of 86 patients with baseline 448-gene or 10-gene NGS data, 15 patients were from the aggressively progressive group, while other 71 patients were from the slowly aggressive group. The OncoGrid depicts frequencies including a composite of all alterations (Intronic, nonFrameShift mutation, nonMissense mutation, FrameShift mutation and Missense mutation). Gene mutation status revealed that concomitant mutations were recombinant tumor protein 53 (TP53) (37.2%), lpha spectrin (SPTA1) (8.1%), lipoprotein receptor-related protein 1B (LRP1B) (8.1%), and retinoblastoma 1 (RB1) (8.1%) (Fig. 2). Other concomitant mutations in aggressively progressive patients but not slowly progressive patients included AXIN2 (13.33%, *n* = 2), PLCG2 (13.33%, *n* = 2) and RAD51C (13.33%, *n* = 2) mutations (Fig. 3). There is significance of those mutations (*P* = 0.029). Therefore, we suspect that those mutations might be an important factor in advanced EGFR-mutant NSCLC patients that demonstrate poor responses to first-line TKI treatment. It requires further investigation in a larger patient cohort.

### Assessment of the Likelihood of Ki-67 and first-line targeted therapy

**Fig. 4:**
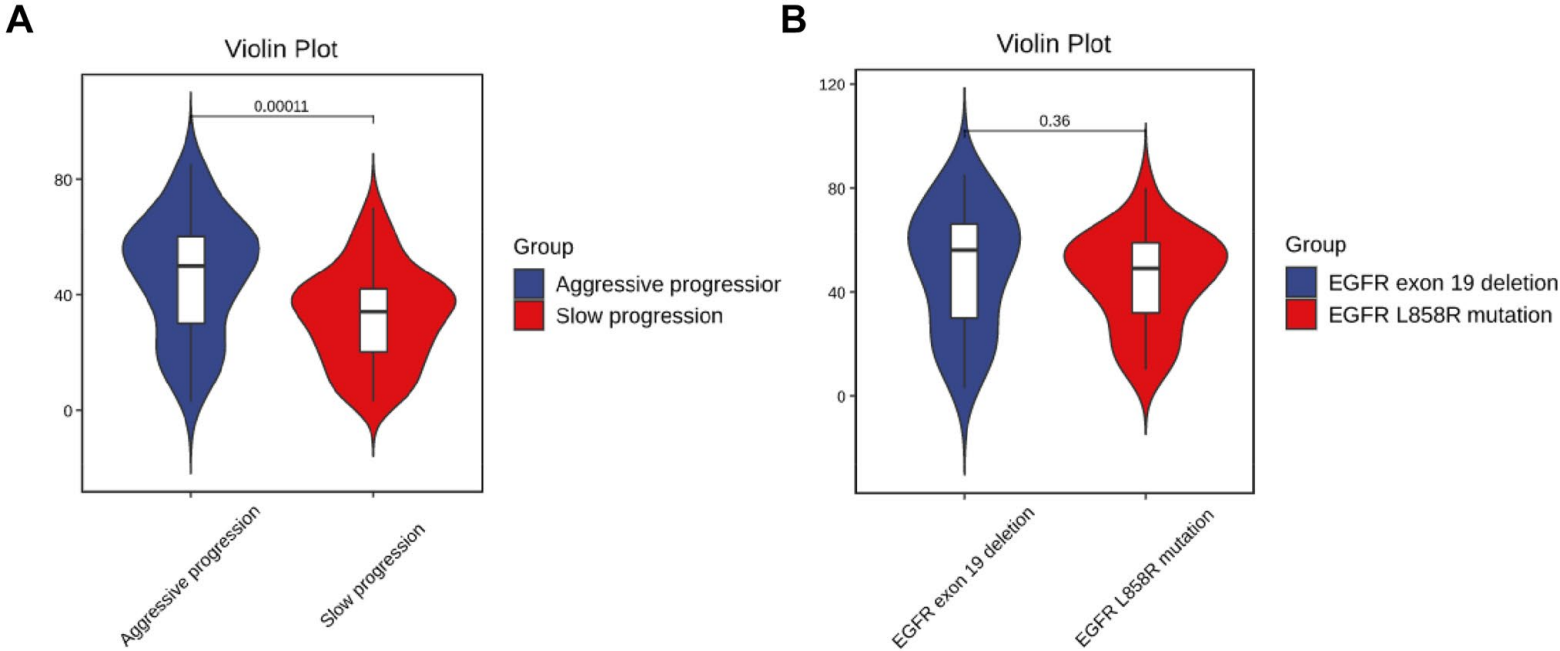
Ki-67 expression according to the response of first-line EGFR TKI treatment (**A**) and subtypes of EGFR mutations in the aggressively progressive group (**B**)

In the analysis, we found that Ki-67 had higher expression level in the aggressively progressive group, and there was significantly statistical difference between those two groups (*P* < 0.05) (Fig. 4A). But according to the subtypes of EGFR mutations in the aggressively progressive group, we revealed that there was no statistical difference in Ki-67 expression between the EGFR 19del group and EGFR L858R mutation group in the aggressively progressive group (*P* = 0.36) (Fig. 4B).

**Fig. 5:**
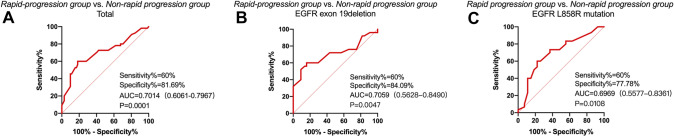
The area under the receiver operating characteristic (ROC) curve using the Ki-67 index to respectively predict the accuracy of the therapeutic effect of first-line EGFR TKI treatment is shown in the entire experimental group (**A**), the EGFR exon 19 deletion group (**B**), and the EGFR L858R mutation group (**C**). AUC, area under the curve

**Fig. 6:**
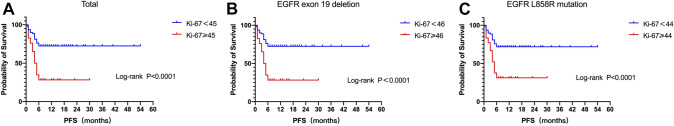
Kaplan–Meier curves for progression-free survival (PFS) of first-line EGFR TKI according to the Ki-67 high expression group and the Ki-67 low expression group. **A** Overall population; **B** EGFR 19del mutation; **C** EGFR L858R mutation

Based on the differences in Ki-67 expression, we applied a receiver operating characteristic (ROC) curve analysis to predict how effective the Ki-67 index was when it was used to judge efficacy of first-line EGFR TKI treatment, and we found that Ki-67 does reflect efficacy. In all subjects, the sensitivity was 60% and the specificity was 81.69% [*P* < 0.01, area under the curve (AUC) = 0.701, 95% confidence interval (CI): 0.606–0.797]. In subgroups of EGFR mutations, the sensitivity was 60% and the specificity were respectively 84.09% and 77.78%. [In the EGFR 19del group, *P* = 0.005, area under the curve (AUC) = 0.706, 95% confidence interval (CI): 0.563–0.849; In the EGFR L858R group, *P* = 0.011, area under the curve (AUC) = 0.697, 95% confidence interval (CI): 0.558–0.836] (Fig. 5). In the overall group, the best cut-off value for the Ki-67 index was 45% for the effective TKI group and the poor efficacy group. In the EGFR 19del group and L858R group, the best cut-off value for the Ki-67 index was respectively 46% and 44% for the effective TKI group and the poor efficacy group. All patients were divided into two groups according to the cut-off value of the Ki-67 index and we found that the lower Ki-67 level, the better PFS time of the first-line TKI therapy (Fig. 6).

To find out which factors affect the survival time of first-line treatment, the univariate and the multivariate Cox proportional hazards regression model was conducted. It showed that Ki-67 was a hazardous factor significantly influencing PFS of the first-line TKI therapy (*P* < 0.05) (Table 2). Compared with patients with Ki-67 < 45%, patients with Ki-67 ≥ 45% showed a faster rate of progression within 6 months.

**Table 2:**
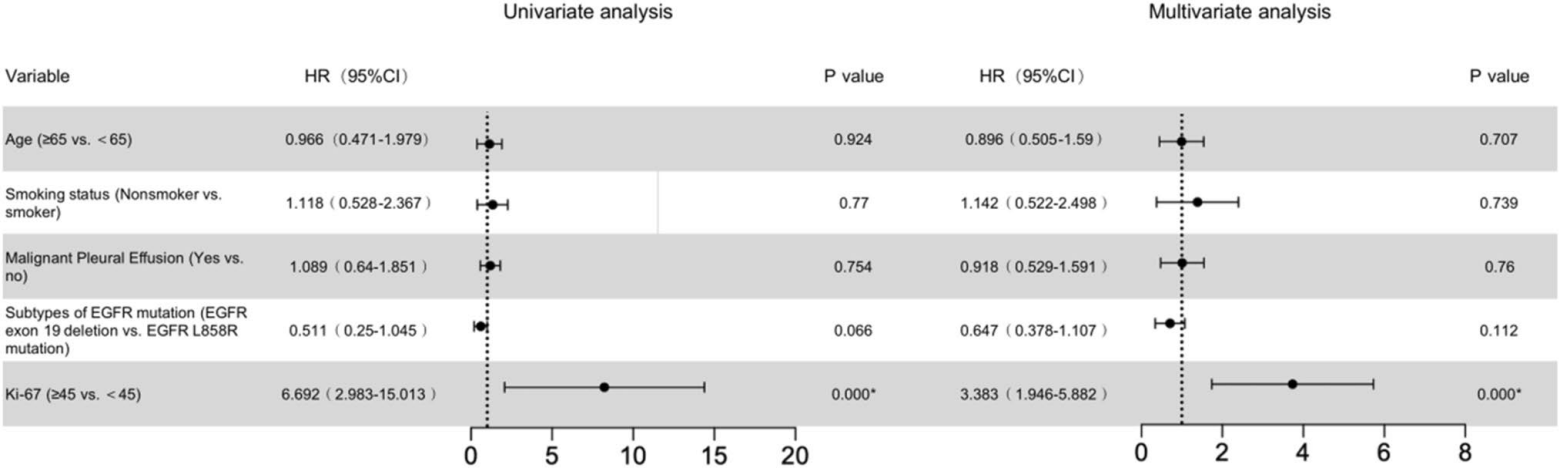
Prognostic factors in the Cox proportional hazards model

CI, confidence interval; HR, hazard ratio

*P < 0.05

### PFS for first-line targeted therapy

**Fig. 7:**
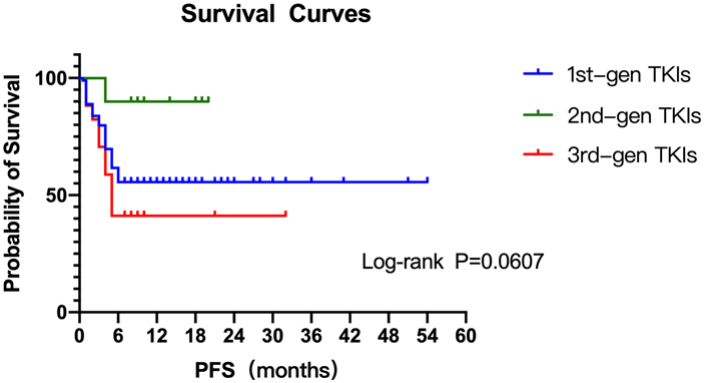
Kaplan–Meier curves for progression-free survival (PFS) according to the different generations of first-line EGFR-TKI groups

Possibly due to historical reasons, among the 126 enrolled patients, 98 (77.78%) received first-generation TKIs for first-line treatment, while only 10 (7.94%) received second-generation TKIs and 18 (14.28%) received the third-generation TKIs. Then we applied Kaplan–Meier curves analysis and found that second-generation TKIs might have a better PFS time on the first-line treatment, even though there was no statistical difference (*P* = 0.0607) (Fig. 7).

### Distribution and PFS of second-line TKI therapy in the aggressively progressive patients

**Fig. 8:**
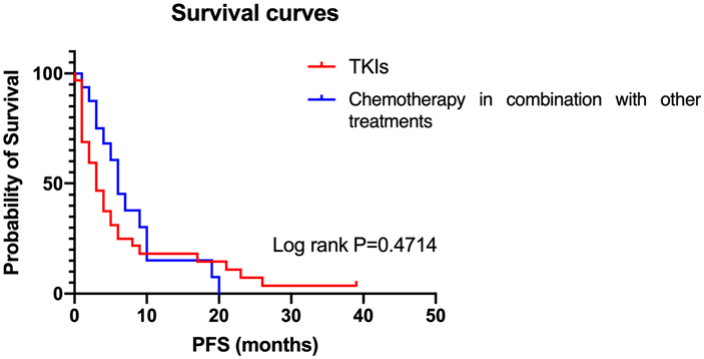
Second-line therapy in the aggressively progressive group. Kaplan–Meier curves for progression-free survival (PFS) according to the different therapy groups of second-line treatment

For those patients who showed aggressive progression on first-line therapy, the choices of second-line treatment seems particularly important. In the aggressive group, 65% of patients continued TKI therapy as a second-line treatment option and 21% of them received chemotherapy combined with EGFR-TKI. Then we applied Kaplan–Meier curves analysis according to the various therapies of second-line treatment, which showed that PFS of chemotherapy in combination with other treatments was better than single TKIs during the first 10 months (Fig. 8), which might be due to the small sample size. We also learned that the median survival time of chemotherapy in combination with other treatments is better than single TKIs (mPFS: EGFR-TKI: 3 months; Chemotherapy combined: 6 months) (*P* = 0.4714).

## Discussion

In our study, we analyzed 55 patients with aggressive progression from a cohort of 1073 cases, which were classified into two groups based on the PFS duration of first-line TKI treatment. According to the depicts of the OncoPrint, 20% of patients (2 out of 10) in the aggressively progressive group respectively carried AXIN2, PLCG2 or RAD51C mutation. To our knowledge, AXIN2, a component of the Wnt signaling pathway, plays an important role in tumor suppression. AXIN2 mutations may contribute to abnormal activation of the Wnt signaling pathway and enhance the proliferation and invasion of tumor cells (Li et al. 2015). Accumulated evidence suggests that AXIN2 has close relations with the development of colon cancer, breast cancer, liver cancer and so on (Liu et al. 2000; Dai et al. 2019; Dong et al. 2019). Zhang et al. (Zhang et al. 2022) conducted targeted sequencing with the 1000-gene panel in NSCLC patients treated with EGFR TKIs and found that AXIN2 might be one of the key genes to the prognosis of NSCLC treated with EGFR TKIs. We also found AXIN2 mutation in only two patients whose PFS for first-line treatment lasted only 1–2 months. We assumed that AXIN2 might be one of the factors contributing to the aggressive progression of first-line TKI therapy.

Phospholipase Cγ2 (PLCG2) is a critical signaling molecule and associated with cancer, neurodegeneration and immune disorders (Jackson et al. 2021). Ma et al. (Ma et al. 2019) concluded that PLCG2 might promote hepatocyte proliferation in rat liver cell via ERK and NF-κB pathway. In small cell lung cancer (SCLC), Chan et al*.* (Chan et al. 2021) found a phenotype with high PLCG2 expression, which was stem-like and significantly correlated with metastases and worse overall survival. In recent years, clinical researchers have identified PLCG2 as a tumor microenvironment (TME)-related gene in soft tissue sarcoma (Li et al. 2021a). They argued that high expression of PLCG2 might help form anti-tumor TME via IL-6/JAK/STAT3 signaling pathway, and then promote the proliferation, invasion and metastasis of tumor cells. However, few studies have described the roles of PLCG2 in NSCLC. Based on the above reports and our study, it is reasonable to believe that PLCG2 may stimulate rapid proliferation and metastasis of tumor cells by remodeling tumor microenvironment or activating specific signaling pathways, which contributes to poor efficacy of EGFR TKI treatment.

RAD51C, a newly identified gene highly associated with breast and ovarian cancer, is involved in the DNA double-strand break repair pathway and plays an important role in DNA damage response. Chen et al*.* (Chen et al. 2016) investigated the clinical significance of RAD51C in NSCLC and suggested that high RAD51C might be an independent predictor of poor efficacy in NSCLC patients receiving chemotherapy and/or radiotherapy. However, the effect of RAD51C in NSCLC patients receiving EGFR TKI has not been reported. More and more in-depth studies are needed in the future to clarify the relationship between RAD51C and efficacy of EGFR TKI in NSCLC.

In addition to compound mutations, we were looking for other predictors. Ki-67 is widely considered as a marker of proliferation because it is expressed in all phases of the cell cycle except the G0 phase (Folescu et al. 2018). Ki-67 has significant prognostic value in breast cancer (Yerushalmi et al. 2010) and prostate cancer (Pollack et al. 2004), and high Ki-67 is associated with poor prognosis in early breast cancer and prostate cancer. Currently, plenty of studies have analyzed the potential role of high Ki-67 index in NSCLC. Most studies have focused on patients with early-stage NSCLC (stage I–III), and a meta-analysis have shown that high Ki-67 index predicts worse DFS and OS in patients with early-stage NSCLC (Xu et al. 2019). At the same time, because NSCLC is a cancer with different histological subtypes, different histological subtypes of patients may have different clinical outcomes. Ki-67 might be an independent prognostic factor for lung adenocarcinoma (Li et al. 2021b), but not all analyses have statistical differences.

In terms of predicting efficacy, Ki-67 index might predict the efficacy of chemotherapy. Wang et al*.* (Wang et al. 2020) reported that the high expression of Ki-67 may also be an indicator of the shortened PFS of chemotherapy. Nevertheless, studies that have used Ki-67 index to predict the efficacy of targeted therapy in advanced NSCLC are rare. In our study, we found that high expression of Ki-67 (Ki-67 ≥ 45%) may be an indicator of poor efficacy of first-line EGFR TKI treatment and its specificity is in the range of 77–84%. We argued that Ki-67 might be used as a biomarker to predict first-line EGFR TKI curative treatment in advanced EGFR-mutant NSCLC patients.

Compared with the first-generation EGFR TKI, the second-generation EGFR TKI can inhibit multiple ErbB family members (EGFR/ErbB1, HER2/ErbB2, ErbB3 and ErbB4) simultaneously and effectively interrupt downstream information transmission (Park et al. 2016), while the third-generation EGFR TKI can irreversibly and selectively inhibit both EGFR-TKI-sensitizing and EGFR p.Thr790Met (T790M) resistance mutations. The efficacy of single EGFR TKI is various in different populations and different gene mutation types, requiring more refined management of patients. FLAURA study compared the third-generation EGFR TKI with the first-generation EGFR TKI and demonstrated a significant benefit in OS, but unfortunately there was no statistical difference between the Asian population and exon 21 L858R mutation (Ramalingam et al. 2020). In the ARCHER 1050 study, on the contrary, Asian populations and patients with exon 21 mutations benefited from the second-generation drug (Mok et al. 2021). On the other hand, if we focused on the survival data of the exon 21 L858R mutation subgroup alone, the OS in this subset of patients in the ARCHER 1050 was nearly 10 months, which is longer than the first-generation EGFR TKI (Mok et al. 2021). In FLAURA, patients with exon 21 mutations had the third-generation and the first-generation EGFR TKI with no statistical difference between the two groups (Ramalingam et al. 2020). Therefore, for advanced NSCLC patients with EGFR mutations, especially exon 21 L858R mutation, the choice of the second-generation EGFR TKI may be more reasonable for the first-line therapy.

The exploration of treatment strategies after EGFR TKI aggressive progression is still ongoing. In EGFR-mutant patients with systemic progression, the option of second-line treatment depends on EGFR T790M status. At the time of progression of erlotinib, gefitinib or afatinib, the standard treatment is osimertinib for patients with EGFR T790M mutation (Lim et al. 2018). Combinations of EGFR-TKIs with different drugs (including other TKIs, monoclonal antibodies and chemotherapy) are currently under investigation. An analysis showed that a combination of osimertinib with pemetrexed or cisplatin can delay the emergence of resistance (Monica et al. 2019). But some clinical trials argued that compared with EGFR-TKI plus chemotherapy, the PFS of single TKI was no significant difference, and the OS is even lower when compared with chemotherapy in T790M patients (Uchibori et al. 2018; Mok et al. 2017). Immunotherapy with PD-1/PD-L1 inhibitors has become a new standard for second-line treatment of NSCLC. In the IMpower 150 trial, benefit was observed from the addition of atezolizumab and bevacizumab to platinum doublet chemotherapy (the ABCP regimen) in the EGFR-mutant patients. Overall survival and PFS were significantly improved in the ABCP arm, suggesting immunotherapy combined with antivascular therapy or combined chemotherapy are potential therapeutic strategies (Passaro et al. 2021). In our study, we found that for the aggressively progressive patients, PFS of chemotherapy in combination with other treatments was better than single TKI during the first 10 months in the second-line therapy. Although the difference was not significant between two groups, the tendency remained. Therefore, the combination therapy to overcome first-line-TKI aggressive resistance needs further confirmation. Genetic profiling of the tumor at aggressive progression could help to identify of resistance mechanisms and select the most appropriate combination approach.

Several limitations must be noted in our study. The samples we selected for this study were limited. We delimited the cut-off value of Ki-67 according to ROC curves, but since many individual studies have defined different cut-off value of Ki-67, it may lead to the heterogeneity of observation results. Future prospective studies are needed to confirm the cut-off value of Ki-67 and the relationship between Ki-67 and prognosis in advanced NSCLC. Last but not least, gene variation is one of the important factors affecting the efficacy of first-line targeted therapy in NSCLC patients. Since most patients in our current study did not complete the 448-gene panel after aggressive progression, we could not identify the possible mechanism leading to aggressive progression.

In conclusion, we observed that high expression of Ki-67 might be a possible indicator of short-term survival on first-line targeted therapy. Additionally, for those patients with high Ki-67 expression (≥ 45%) or combined with AXIN2, PLCG2 or RAD51C mutation, we may predict that they are likely to show aggressive progression on first-line EGFR TKI therapy. And for these patients, second-generation TKI may benefit more from first-line targeted therapy. When they show aggressive progression on first-line TKI therapy, we argued that combination therapy might be considered instead of single TKI therapy.

## Data Availability

The datasets generated during and/or analyzed during the current study are available from the corresponding author on reasonable request.
